# 肿瘤分子标志的发现和验证：问题和策略探讨

**DOI:** 10.3779/j.issn.1009-3419.2010.12.01

**Published:** 2010-12-20

**Authors:** 雨晨 李, 韦 宋

**Affiliations:** 1 100038 北京，首都医科大学肿瘤学系，北京世纪坛医院肿瘤研究室 Cancer Research Laboratory, Beijing Shijitan Hospital; Department of Oncology, the Capital Medical University, Beijing 100038, China; 2 100142 北京，北京肿瘤医院，北京肿 瘤防治研究所，北京大学临床肿瘤学院，恶性肿瘤发病机制及转化研 究教育部重点实验室，临床研究实验室 Key Laboratory of Carcinogenesis and Translational Research of Ministry of Education, Clinical Research Laboratory, Peking University School of Oncology, Beijing Cancer Hospital & Institute, Beijing 100142, China

**Keywords:** 肺肿瘤, 肿瘤标志物, 诊断, Lung neoplasms, Tumor biomarker, Diagnosis

## 1 现状和问题分析

分子标志在肿瘤筛查和诊断、预测疗效、复发、转移及预后方面呈现巨大潜力。同时，许多分子标志本身也是治疗肿瘤的药物靶点。这一认识很大程度归功于我们对肿瘤在分子水平上认识的极大丰富和高通量芯片新技术的不断产生和更新。Hanahan和Weinberg博士^[1]^在2000年提出肿瘤细胞6大生物学特征：①逃逸凋亡信号；②过度生长信号；③对生长抑制信号不敏感；④复制潜能无限；⑤血管生成持续；⑥组织侵袭和转移。加之近年来对肿瘤细胞赖于生存的微环境重要性认识的加强，组成了“肿瘤6+1生物学特征”的新理念。肿瘤分子标志的发现源于这“6+1生物学特征”，与肿瘤临床特性密切相关，成为肿瘤筛查、诊断和病程监控的极富潜力的新手段。

于是，早在1996年美国NCI等几家著名肿瘤研究机构牵头制定了分子标志肿瘤临床应用价值的评价体系^[2]^（表 1）。按6-档评分制，根据分子标志与生物学特征和临床终止点的相关性对其是否具有临床应用价值进行评分。只有得分为++和+++的分子标志才能用于常规肿瘤的临床检测，协助制定临床决策，其可能是临床决策唯一或非唯一的标准。

**1:** 肿瘤标志临床应用评分细则

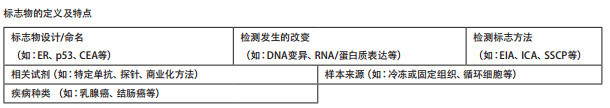												
应用												
	与生物学特征相关				与临床终止点相关性							
	过程		终点		总生存期		无病生存期		生活质量		治疗成本	
应用	应用水平	应用评分	应用水平	应用评分	应用水平	应用评分	应用水平	应用评分	应用水平	应用评分	应用水平	应用评分
风险测定												
筛查												
鉴别诊断												
预后：预测复发/进展												
原发												
转移												
预后：预测治疗效果												
原发												
转移												
监控												
监控治疗后无症状患者复发												
跟踪可检测的疾病												
ER：雌激素受体；CEA：癌胚抗原；FNA：细针穿刺活检术；EIA：酶联免疫反应；ICA：免疫化学反应；SSCP:单链构象多态性^[2]^。												

2000年，美国临床肿瘤协会（American Society of Clinical Oncology, ASCO）肿瘤标志专家组更新了1997年推荐的分子标志用于结直肠癌和乳腺癌临床实践的指南，但推荐更新的内容与1997年相比无明显差异^[3]^。*CEA*、*CA-199*、*p53*和*Ras*基因虽然用于结直肠癌检测，但由于特异性和敏感度低等原因仍不推荐用于筛查和诊断分期。CE A仅推荐用于治疗前后每2-3个月跟踪监测结直肠癌的进展情况。用于乳腺癌的肿瘤标志主要是雌激素受体（estrogen receptor, ER）、孕激素受体（progestogerone receptor, PR）和HER-2/neu。对停经前后的患者，推荐检测ER和PR用以挑选最可能对激素类辅助治疗敏感和治疗复发或转移的患者。检测*HER-2/neu*基因扩增或表达可用于挑选对曲妥珠单抗（Herceptin）有效的转移、复发或手术无法切除的局部晚期患者。

然而，10年已经过去。与2000年相比，至今应用于肿瘤临床的分子标志仍寥寥无几。为什么20年的肿瘤标志研究和数百篇的研究报道（尤其是近10年）没有产生更加理想的结果？这可能很大程度归咎于我们研究报道的实验结果在前瞻性临床试验验证中难以重复。

首先，在分子标志的发现和验证阶段存在问题。研究方法、实验设计、检测手段、样本和数据收集和处理、统计分析方法等方面存在任何的不合理性都可能影响实验结果。CEA是典型例子，反映了样本收集标准不一致带来的后果：1969年PNAS的文章^[4]^报道CEA用来筛查结直肠癌的敏感性和特异性接近100%。随后在10年的应用中发现，CEA筛查结直肠癌的敏感性和特异性远远不如最初报道的结果。1978年NEJM的文章^[5]^分析其原因主要在于发现和验证时采用的肿瘤样本恶性程度高，而随后用于筛查时，面对的大多是无症状或早期肿瘤。

## 2 共识和策略探讨

据此，国际肿瘤研究和临床学界对分子标志在发现和验证阶段存在的问题展开讨论，达成共识，以引导和规范化肿瘤分子标志发现和验证的研究^[6, 7]^。

### 2.1 发现阶段存在的主要问题

（1）患者选择标准不统一（如：切除程度、是否辅助治疗）；（2）样本收集和处理不一致（如：快速冷冻或方便获得的组织）；（3）多因素分析漏掉NCCN指南的多项临床风险因子、未比较它们之间优劣势（如：切除程度、肿瘤大小）；（4）未做亚级分析或样本小量，分析结果不准，难以与临床指标联合使用，提高预测力；（5）建立预测模型细节不详（如：权重和阈值）或漏掉临床指标（如：肿瘤大小）；（6）标志物模型能预测的一组患者，临床指标也能预测，不呈现优势；（7）忽略重要临床需求（如：挑选出ⅠA级需要化疗的高复发风险的患者、ⅠB级和Ⅱ级不需要化疗的低复发风险的患者）。

### 2.2 验证阶段存在的主要问题

#### 2.2.1 体制问题

（1）评价诊断和预后分子标志的体系和规则远不如评价治疗方案的体系和规则完善。评估治疗方案采用实验性方法，如随机对照临床试验，能有效地处理异源性、复杂性和偏见性等对结果验证的威胁；而评价诊断和预后采用观察或临床流行学的非实验性方法，且验证程序粗糙。（2）FDA等监督管理部门对药物研发和市场的监督力度也促进评价治疗方案的规章的完善，而对诊断和预后研究却没有相关监督管理部门。

#### 2.2.2 方法问题

（1）验证方法多样，如随机取样验证、样本分割验证及样本分割交叉验证（如：留一交叉验证法）；（2）验证集样本小（< 50）或样本大但异源性太高，与训练集差异太大；（3）独立验证仅占研究报道的10%，更缺乏“外来样本”的独立验证；（4）多个标志在小样本中发现和验证，造成统计学上的Overfit现象。

## 3 针对肿瘤标志研究的建议性指南

针对这些问题，美国NCI等相关专家提出针对肿瘤标志研究的建议性指南^[6]^，主要包括：（1）明确研究目的，尤其是临床研究目的；（2）对数据收集和报道，要求：①谨慎地设计患者入组和排除标准及样本量，②准确报道患者治疗方案和所有常规临床指标，③详细描述样本收集方法和实验手段，④公布所有原始实验数据；（3）统计分析和结果报道，要求：①详细描述统计方法，包括数据标准化方法、数据过滤方法、变量选择、建模技术、丢失数据处理、阈值选择及其合理性，②终止点是死亡或复发时间，数据处理不应该进行二进制变换，③结果至少在一组完全独立的样本中验证，④证明标志物的临床应用价值；（4）详细描述预测模型的建立，以便其他研究者可以用独立样本验证模型；（5）与现有手段比较，明确提出新结果的优势及局限性。

此次，研究结果的报道方式也存在问题。报道方式不严密、不统一，导致难以有效地评价、比较和验证研究质量和研究结果。为此，NCI-EORTC对标志物研究（尤其预后标志研究）的论文提出审稿标准20条的建议，其目的是便于评价研究设计、方法和结果分析，提高不同来源的研究结果间的可比性，增强研究透明性和研究工作的完整报道^[8]^。表 2列出审稿标准20条建议。

**2:** 肿瘤标志研究审稿标准20条建议^[8]^

肿瘤标志物研究指南
引言：
1.阐明被检测的标志物的名称、研究目的及假说。
材料和方法：
患者：
2.描述入组患者特征（疾病分期或合并症），包括患者来源、入组及排除标准。
3.描述患者的治疗方式及入组治疗方式的选择（如：随机或根据规则入组）。
样本特征：
4.描述使用的生物样本（包括对照组）及处理和贮存的方法。
检测方法：
5.详细介绍研究中使用的检测方法并提供（或参考）详尽的实验流程，包括实验用到的特殊试剂或试剂盒、质量控制程序、重复性评价、量化方法及对实验流程的评价和报道。指明实验方法如何做到以及是否为盲终点研究。
实验设计：
6.阐明选择患者入组的方法，包括实验设计是否是前瞻性、回顾性及是运用分层还是配对进行分析（例如：是利用疾病分级还是年龄进行分析）。详细说明患者入组时间、随访结束时间以及中位随访时间。
7.准确定义所有研究中所涉及的临床终点事件。
8.列出所有可能的被第一次检测或考虑的列入模型的变量。
9.给出样本大小的合理性依据。数据分析方法：
10.详细阐明研究中运用的统计学方法，包括任何一种变量选择程序的细节以及模型建立的问题，模型的假设是如何被证实的以及丢失的数据是如何处理的。
11.阐明标志物的值在分析中是如何处理的，并说明确定阈值的方法。
结果：
数据：
12.描述患者参与此项研究的过程（流程图），包括各个研究分析阶段所需患者的人数以及退组的原因。特别要注明在分组或全组详细的检查报告中患者人数和发生的事件。
13.说明基本的人群分布特征（年龄和性别）、标准化的（具有疾病特殊性的）预后变量以及肿瘤标志物，包括丢失数据的数量。
数据分析及陈述：
14.陈述检测标志物与标准的临床预后变量间的关系。
15.单因素分析标志物与临床转归的关系，并加入估计的效率（如：风险阈值和生存可能性）。最好也提供其它变量的类似的相关性分析结果。对于评价肿瘤标志物对于时间性事件的结果的影响（如，预后），应进行*Kaplan-Meier*分析。
16.对关键性的多因素分析，应在置信区间内报道标志物的估计效率（如：风险阈值），并且至少在最终模型中加入所有其它变量一起分析。
17.在所有报道的结果中，对标志物和标准临床预后变量分析时提供有置信区间的估计效率，不管它们是否有统计学上的重要性。
18.若进行进一步分析，如检查假设、敏感度分析和内部验证，应报道所有实验结果。
讨论：
19.结合预设的假说和其它研究报道，讨论和解释研究结果，包括本研究的局限性。
20.讨论此项研究对今后研究和临床价值的潜在价值。

## 4 结语

至今，分子标志在临床应用最成功的方面应该是用于指导个体化靶向治疗。如：检测*Her2/neu*基因扩增或表达，用于指导挑选对靶向药曲妥珠单抗敏感的乳腺癌患者，*EGFR/k-Ras*突变检测指导挑选对吉非替尼（Iressa）和厄洛替尼（Tarceva）敏感的肺癌和结直肠癌患者。希望未来的10年，在更加严谨的实验设计、实验操作和完善的评价机制引导下，更多有潜力的分子标志被推向临床应用。

## References

[1] Hanahan D, Weinberg RA (2000). The hallmarks of cancer. Cell.

[2] Hayes DF, Bast RC, Desch CE (1996). Tumor marker utility grading system: a framework to evaluate clinical utility of tumor markers. J Natl Cancer Inst.

[3] Bast RC Jr, Ravdin P, Hayes DF (2001). 2000 update of recommendations for the use of tumor markers in breast and colorectal cancer: clinical practice guidelines of the American Society of Clinical Oncology. J Clin Oncol.

[4] Thomson DM, Krupey J, Freedman SO (1969). The radioimmunoassay of circulating carcinoembryonic antigen of the human digestive system. Proc Natl Acad Sci USA.

[5] Ransohoff DF, Feinstein AR (1978). Problems of spectrum and bias in evaluating the efficacy of diagnostic tests. N Engl J Med.

[6] Subramanian J, Simon R (2010). Gene expression-based prognostic signature in lung cancer: ready or clinical use. J Natl Cancer Inst.

[7] Ransohoff DF (2004). Rules of evidence for cancer molecular-marker discovery and validation. Nat Rev Cancer.

[8] McShane LM, Altman DG, Sauerbrei W (2005). Reporting recommendations for tumor marker prognostic studies. J Clin Oncol.

